# Supplementary material for: References to unbiased sources increase the helpfulness of community fact-checks
Source: Sci Rep. 2025 Jul 16;15:25749. doi: 10.1038/s41598-025-09372-6 (PMC12267575; doi:10.1038/s41598-025-09372-6)
Supplement: Supplementary file 1 — Supplementary Information. [file 41598_2025_9372_MOESM1_ESM.pdf]

Supplementary Information

Contents

A Summary Statistics 2

B Cross-Correlations 3

C Categorization of Media Types 4

D Topic Prediction 5

E Analysis with Factuality Scores and Domain Quality Scores 6

F Analysis of Total Votes 6

G Analysis Without Random Effects 6

H Estimation Results 8

A Summary Statistics

Supplementary Table S1 provides summary statistics for the variables in our analysis. On average, each Community Note in our sample received 69.267 votes (helpful and unhelpful) from users with a standard deviation of 149.572 votes. Out of these, $66.60\,\%$ of all votes categorized the Community Note as being helpful. Among all Community Notes in our dataset, approximately $88.66\,\%$ contain at least a single link to an external website. The mean bias of the external source is 0.726. However, on average, the links provided in Community Notes tend to be more left-leaning. Specifically, *Source Bias (Left)* is present in approximately $52\,\%$ of notes, followed by *Source Bias (Undirected)* with approximately $34\,\%$, and *Source Bias (Right)* with approximately $14\,\%$.

We also observe substantial heterogeneity regarding the control variables in our analysis. On average, an author of a fact-checked posts has $5{,}839{,}784$ followers and $11{,}299$ followees. Approximately $63\,\%$ of the original post's authors are verified. The account age ranges from 5.05 months to 17.31 years with a mean of 8.99 years. Slightly less than half ($42.1\,\%$) of the fact-checked posts covers a political topic. The age of the original posts in our dataset ranges from 17 days to 11.54 years, with a mean value of under half a year (109 days). The sentiment of the Community Notes is, on average, slightly negative with a mean sentiment of -0.033. Similarly, the sentiment of the original posts is slightly negative as well (mean of -0.037). The average length of a Community Note is 31.747 words, and the mean text complexity (Gunning-Fog index) is 14.01.

Variable	Mean	Median	Min	Max	SD
DEPENDENT VARIABLES					
HVotes	46.130	18.000	0.000	5145.000	118.663
Votes	69.267	31.000	1.000	5609.000	149.572
INDEPENDENT VARIABLES					
Fact-Check					
External Source	0.887	1.000	0.000	1.000	0.317
Bias Magnitude	0.726	1.000	0.000	2.000	0.579
Bias Direction (Left)	0.520	1.000	0.000	1.000	0.500
Bias Direction (Center)	0.344	0.000	0.000	1.000	0.475
Bias Direction (Right)	0.136	0.000	0.000	1.000	0.343
Word Count	31.747	34.000	0.000	91.000	12.052
Text Complexity	14.010	13.689	0.400	40.800	4.935
Sentiment	−0.033	−0.014	−0.989	1.418	0.200
Original post					
Followers (in 1000s)	5839.919	232.726	0.000	144,605.508	22,713.203
Followees (in 1000s)	11.299	1.123	0.000	1486.542	56.968
Verified	0.625	1.000	0.000	1.000	0.484
Account Age	8.989	10.489	0.058	17.307	5.152
Political	0.421	0.000	0.000	1.000	0.494
Post Age (in days)	109.200	84.801	16.952	4612.346	123.925
Post Sentiment	−0.037	−0.021	−1.000	2.000	0.232

Note: Bias ratings are only available on the subset of Community Notes containing links to at least one external website.

Supplementary Table S1: **Descriptive Statistics**

B Cross-Correlations

Cross-correlations among the independent variables are reported in Supplementary Fig. S1.

We observe weak positive correlations between *Followers* and *Verified* (corr: 0.193; $p < 0.001$), and in addition, between *Account Age* and *Verified* (corr: 0.085; $p < 0.001$). This can be expected as larger and older accounts are more likely to have already been verified by X (and vice versa). In line with this, we also find a weak positive correlation between *Followers* and *Account Age* (corr: 0.185; $p < 0.001$). Additionally, we observe weak positive correlations between *Text Complexity* and *Word Count* (corr: 0.274, $p < 0.001$). We also observe a weak positive correlation between *Account Age* and *Political* (corr: 0.049, $p < 0.001$), as well as between *Verified* and *Political* (corr: 0.052, $p < 0.001$), which may be explained by the fact that larger and more prominent accounts are more likely to be verified (e. g., almost every member of the U. S. Congress is verified [1]). Furthermore, there are weak positive correlations between *External Source* and *Word Count* (corr: 0.149, $p < 0.001$), and in line with it, weak positive correlation between *External Source* and *Text Complexity* (corr: 0.124, $p < 0.001$). Lastly, there is a weak negative

correlation between *Post Sentiment* and *Political* (corr: -0.028, $p < 0.001$) All remaining correlations are rather small.

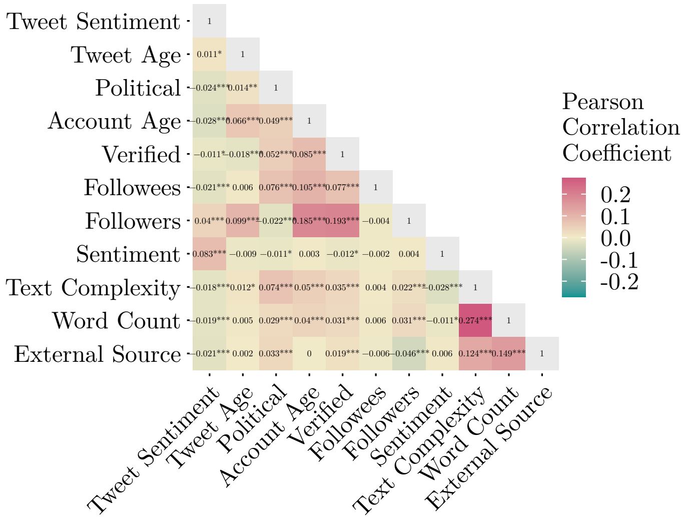

Supplementary Figure S1: Cross-Correlations Among Independent Variables.

C Categorization of Media Types

We instructed two trained research assistants to manually categorize all domains linked to in Community Notes into seven predefined media categories: *Media Outlets* (e. g., CNN), *Public Authorities* (e. g., CDC), *Social Media* (e. g., X), *Encyclopedias* (e. g., Wikipedia), *Third-Party Fact Checkers* (e. g., Snopes), *Scientific Literature* (e. g., Nature), and *Other* (i. e., domains that do not fall into one of the other categories; e. g., links to Google Maps). These media categories have been identified based on a manual assessment of the links included in Community Notes.

The most common sources in Community Notes are links to *Media Outlets* and *Public Authorities*, which represent $50.09\,\%$ and $18.25\,\%$ of all links in our dataset respectively. This is followed by links to *Social Media Posts* ($13.97\,\%$), *Scientific Literature* ($7.04\,\%$), *Encyclopedias* ($5.82\,\%$), and *Third-Party Fact Checkers* ($3.34\,\%$). Only $1.50\,\%$ of the links could not be assigned to at any of these categories.

D Topic Prediction

We implemented a supervised machine learning model to classify whether fact-checked posts cover specific topical domains. Supervised learning approaches are known to significantly outperform unsupervised methods (e. g., Latent Dirichlet Allocation, keyword matching) for short texts [2].

Training of machine learning model: We used the TwHIN-BERT (large) model [3] as the base for our classifier. This model was pre-trained on a large corpus of 7 billion posts from X, incorporating interaction-based social objectives to enhance its contextual understanding. We fine-tuned this model on a manually annotated subset of 1500 Community Notes posts. Two trained research assistants independently labeled one half each, with 175 overlapping posts included to measure inter-annotator agreement. This yielded a macro-averaged Cohen's κ of 0.711 and an agreement rate of $90.8\,\%$. The model was implemented in Python 3.11.3 using the Transformers library (version 4.30.2).

Topic schema and coverage: The classifier was trained to assign one or more topic labels to each source post based on five predefined categories: *Politics*, *Health*, *Economy*, *Science*, and *Other*. Topic definitions were informed by prior work and refined through manual inspection during annotation. While all five categories were used in extended analyses, our main models focused on identifying political content due to the paper's emphasis on political discourse. The topical categories of *Health*, *Economy*, and *Science* form specific subgroups within the broader *non-political* class used in the main analysis. The full distribution of topic labels, including counts and percentages, is reported in Table S2.

Validation of topic predictions: We evaluated the topic predictions of the machine learning model using 10-fold cross-validation in combination with the manually annotated posts to calculate the out-of-sample prediction performance. Here, the machine learning classifier yielded a high out-of-sample accuracy of 0.904 and an $F1$-score of 0.755.

Topic	Counts (in 1000)	Percentage
Politics	17.322	42.100
Health	6.931	16.900
Economy	6.681	16.200
Science	4.040	9.820
Other	13.757	33.400

Supplementary Table S2: **Distribution of Source Post Topics**

E Analysis with Factuality Scores and Domain Quality Scores

The bias ratings from Media Bias/Fact Check are an aggregate measure based on multiple criteria. In addition, Media Bias/Fact Check provides separate ratings for the level of factual reporting of websites. These ratings are assigned on a six-point Likert scale ranging from -3 (very low) to $+3$ (very high). As a robustness check, we retrieved these factuality ratings and tested a model variant that includes the level of factual reporting of external sources in Community Notes (*Factuality*) as a control variable. Results are reported in Tables S10 and S11. We find that *Factuality* has a positive and statistically significant effect on helpfulness (ME = 0.002, OR = 1.002, $p < 0.001$) when controlling for the level of *Bias Magnitude*. All results from the main paper remain robust and qualitatively unchanged.

As an additional check, we include Domain Quality Scores introduced by [4] as a predictor of perceived helpfulness. Domain Quality Scores represent an aggregate measure derived by applying principal component analysis (PCA) to 16 expert-coded indicators of source reliability. Domain Quality Scores are highly positively correlated with *Factuality* ($r = 0.568$) and negatively correlated with *Bias Magnitude* ($r = -0.312$). The results are reported in Tables S10 and S11. When controlling for *Bias Magnitude*, *Domain Quality* Score is not statistically significant, suggesting that domain quality offers little additional explanatory power for perceived helpfulness beyond the bias of the source. All results from the main paper remain qualitatively unchanged.

F Analysis of Total Votes

As an exploratory analysis, we repeat our regression with the total number of votes (helpful and unhelpful) as the dependent variable. We observe a positive and statistically significant coefficient for *External Source* (coef. = 0.156, IR = 1.169, $p < 0.001$). This implies that Community Notes containing links to external sources receive both a higher total number of votes and a higher share of helpful votes. Full estimation results are reported in Table S15.

G Analysis Without Random Effects

As a further robustness check, we re-estimate our main regression models without fact-checker-specific random intercepts. While some coefficient magnitudes vary slightly, the results are consistent with our main findings. Marginal effects plots and full estimation results are reported below. Full estimation results are reported in Tables S12, S13, and S14.

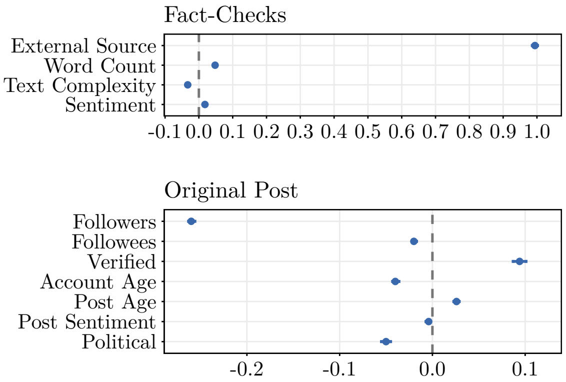

Supplementary Figure S2: Binomial regression analyzing the helpfulness of external sources in explaining the share of helpful votes. Shown are coefficient estimates with **95 %** CIs. Unit of analysis is the fact-check level ($N = 41,129$).

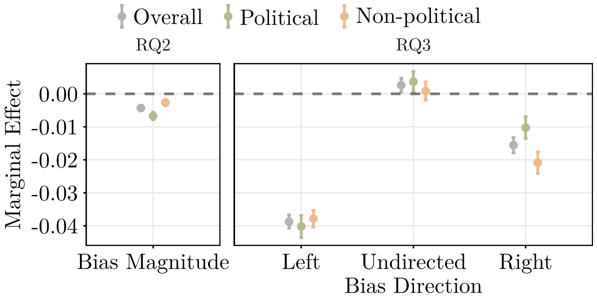

Supplementary Figure S3: Marginal effects (with **95 %** CIs) of bias magnitude (left panel) and bias direction (right panel) on the share of helpful votes. Unit of analysis is the fact-check level ($N = 21,307$).

H Estimation Results

	RQ1
Fact-Check	
Word Count	0.012^{***}
	(0.002)
Text Complexity	-0.040^{***}
	(0.002)
Sentiment	-0.003
	(0.002)
External Source	0.844^{***}
	(0.007)
Original Post	
Followers	-0.223^{***}
	(0.002)
Followees	-0.024^{***}
	(0.002)
Verified	0.155^{***}
	(0.004)
Account Age	-0.017^{***}
	(0.002)
Post Age	0.027^{***}
	(0.002)
Post Sentiment	0.003^{*}
	(0.002)
Political	-0.030^{***}
	(0.004)
Intercept	-0.128^{***}
	(0.013)
Random Effects	Included
AIC	519,979
Observations	41,128

Note: Binomial regression explains the share of helpful votes. Unit of analysis is the fact-check level (N = 41,128). Community Notes that do not link to external sources are excluded. Significance levels: $^{*}p < 0.05$, $^{**}p < 0.01$, $^{***}p < 0.001$. Standard errors are in parentheses.

Supplementary Table S3: **Regression Linking the Presence of External Sources to Helpfulness. RQ1 in the Main Paper.**

	RQ2 Model (1)	RQ2 Model (2)
Fact-Check		
Word Count	0.013^{***}	0.013^{***}
	(0.003)	(0.003)
Text Complexity	-0.064^{***}	-0.065^{***}
	(0.003)	(0.003)
Sentiment	0.009^{***}	0.008^{**}
	(0.002)	(0.002)
Bias in External Sources		
Bias Magnitude	-0.031^{***}	-0.058^{***}
	(0.003)	(0.003)
Politics × Bias Magnitude		0.062^{***}
		(0.005)
Original Post		
Followers	-0.226^{***}	-0.225^{***}
	(0.003)	(0.003)
Followees	-0.024^{***}	-0.025^{***}
	(0.003)	(0.003)
Verified	0.116^{***}	0.114^{***}
	(0.007)	(0.007)
Account Age	-0.004	-0.004
	(0.003)	(0.003)
Post Age	0.005	0.005
	(0.004)	(0.004)
Post Sentiment	-0.020^{***}	-0.020^{***}
	(0.002)	(0.002)
Political	-0.018^{***}	-0.020^{***}
	(0.005)	(0.005)
Intercept	0.776^{***}	0.776^{***}
	(0.015)	(0.015)
Random Effects	Included	Included
AIC	252,168	252,009
Observations	21,307	21,307

Note: Binomial regression explains the share of helpful votes. Unit of analysis is the fact-check level ($N = 21{,}307$). Community Notes that do not link to external sources are excluded. Significance levels: $^{*}p < 0.05$, $^{**}p < 0.01$, $^{***}p < 0.001$. Standard errors are in parentheses.

Supplementary Table S4: **Regression Linking Political Bias in External Sources to Helpfulness. RQ2 in the Main Paper.**

	RQ3 Model (1)	RQ3 Model (2)
Fact-Check		
Word Count	0.013***	0.013***
	(0.003)	(0.003)
Text Complexity	−0.064***	−0.065***
	(0.003)	(0.003)
Sentiment	0.008***	0.007**
	(0.002)	(0.002)
Bias in External Sources		
Bias Magnitude	0.008	−0.005
	(0.007)	(0.010)
Politics × Bias Magnitude		0.035*
		(0.014)
Bias Direction (Left)	−0.039***	−0.071***
	(0.009)	(0.013)
Bias Direction (Right)	−0.013	−0.057**
	(0.012)	(0.018)
Bias Magnitude × Bias Direction (Left)	−0.075***	−0.073***
	(0.010)	(0.013)
Bias Magnitude × Bias Direction (Right)	−0.036***	−0.043**
	(0.010)	(0.014)
Politics × Bias Direction (Left)		0.066***
		(0.019)
Politics × Bias Direction (Right)		0.093***
		(0.024)
Politics × Bias Magnitude × Bias Direction (Left)		−0.009
		(0.019)
Politics × Bias Magnitude × Bias Direction (Right)		0.001
		(0.019)
Original Post		
Followers	−0.227***	−0.225***
	(0.003)	(0.003)
Followees	−0.024***	−0.024***
	(0.003)	(0.003)
Verified	0.117***	0.114***
	(0.007)	(0.007)
Account Age	−0.004	−0.004
	(0.003)	(0.003)
Post Age	0.006	0.006
	(0.004)	(0.004)
Post Sentiment	−0.021***	−0.020***
	(0.002)	(0.002)
Political	−0.019***	−0.066***
	(0.005)	(0.017)
Intercept	0.820***	0.843***
	(0.017)	(0.019)
Random Effects	Included	Included
AIC	252,083	251,918
Observations	21,307	21,307

Note: Binomial regression explains the share of helpful votes. Unit of analysis is the fact-check level ($N = 21{,}307$). Community Notes that do not link to external sources are excluded. Significance levels: $^{*}p < 0.05$, $^{**}p < 0.01$, $^{***}p < 0.001$. Standard errors are in parentheses.

Supplementary Table S5: **Regression Linking Bias Direction in External Sources to Helpfulness. RQ3 in the Main Paper.**

	Model (1)	Model (2)
Fact-Check		
Word Count	0.010***	0.006*
	(0.003)	(0.003)
Text Complexity	−0.063***	−0.060***
	(0.003)	(0.003)
Sentiment	0.010***	0.009***
	(0.002)	(0.002)
Number of External Sources	0.040***	
	(0.003)	
Source Category		
Media Outlets		0.085***
		(0.007)
Public Authorities		0.174***
		(0.008)
Social Media Posts		0.173***
		(0.011)
Encyclopedias		0.000
		(0.009)
Third-Party Fact Checkers		0.265***
		(0.012)
Scientific Literature		0.027*
		(0.011)
Intercept	0.776***	0.685***
	(0.015)	(0.016)
Original Post Controls	Included	Included
Random Effects	Included	Included
AIC	251,898	250,881
Observations	21,277	21,277

Note: Binomial regression explains the share of helpful votes. Unit of analysis is the fact-check level ($N = 21{,}277$). Community Notes that do not link to external sources are excluded. Control variables for the original post are included. Significance levels: $^{*}p < 0.05$, $^{**}p < 0.01$, $^{***}p < 0.001$. Standard errors are in parentheses.

Supplementary Table S6: **Exploratory Analyses and Robustness Checks: Number of External Sources and Topics.**

	Model (1)	Model (2)
Fact-Check		
Word Count	0.013^{***}	0.014^{***}
	(0.003)	(0.003)
Text Complexity	-0.064^{***}	-0.065^{***}
	(0.003)	(0.003)
Sentiment	0.008^{***}	0.007^{**}
	(0.002)	(0.002)
Bias in External Sources		
Bias Left (High)	-0.220^{***}	-0.273^{***}
	(0.014)	(0.017)
Bias Left (Medium)	-0.068^{***}	-0.117^{***}
	(0.006)	(0.008)
Bias Left (Low)	0.038^{***}	-0.006
	(0.010)	(0.013)
Bias Right (Low)	0.068^{***}	-0.162^{***}
	(0.016)	(0.025)
Bias Right (Medium)	-0.065^{***}	-0.112^{***}
	(0.010)	(0.013)
Bias Right (High)	-0.037^{**}	-0.135^{***}
	(0.013)	(0.018)
Politics × Bias Left (High)		0.129^{***}
		(0.028)
Politics × Bias Left (Medium)		0.109^{***}
		(0.011)
Politics × Bias Left (Low)		0.105^{***}
		(0.019)
Politics × Bias Right (Low)		0.387^{***}
		(0.032)
Politics × Bias Right (Medium)		0.108^{***}
		(0.019)
Politics × Bias Right (High)		0.209^{***}
		(0.025)
Source Category		
Intercept	0.817^{***}	0.854^{***}
	(0.016)	(0.016)
Original Post Controls	Included	Included
Random Effects	Included	Included
AIC	$251{,}631$	$251{,}404$
Observations	$21{,}277$	$21{,}277$

Note: Binomial regression explains the share of helpful votes. Unit of analysis is the fact-check level ($N = 21{,}277$). Community Notes that do not link to external sources are excluded. Control variables for the original post are included. Significance levels: $^{*}p < 0.05$, $^{**}p < 0.01$, $^{***}p < 0.001$. Standard errors are in parentheses.

Supplementary Table S7: **Exploratory Analyses and Robustness Checks: Bias Categories.**

	Model (1)	Model (2)
Fact-Check		
Word Count	0.014***	0.014***
	(0.003)	(0.003)
Text Complexity	−0.065***	−0.066***
	(0.003)	(0.003)
Sentiment	0.008***	0.007**
	(0.002)	(0.002)
Bias in External Sources		
Bias Magnitude Medium	−0.043***	−0.104***
	(0.005)	(0.007)
Bias Magnitude High	−0.137***	−0.214***
	(0.009)	(0.012)
Politics × Bias Magnitude Medium		0.136***
		(0.010)
Politics × Bias Magnitude High		0.178***
		(0.018)
Source Category		
Intercept	0.811***	0.851***
	(0.015)	(0.016)
Original Post Controls	Included	Included
Random Effects	Included	Included
AIC	251,859	251,657
Observations	21,277	21,277

Note: Binomial regression explains the share of helpful votes. Unit of analysis is the fact-check level ($N = 21{,}277$). Community Notes that do not link to external sources are excluded. Control variables for the original post are included. Significance levels: $^{*}p < 0.05$, $^{**}p < 0.01$, $^{***}p < 0.001$. Standard errors are in parentheses.

Supplementary Table S8: **Exploratory Analyses and Robustness Checks: Bias Magnitude Categories.**

	Model (1)
Fact-Check	
Word Count	0.012^{***}
	(0.002)
Text Complexity	-0.038^{***}
	(0.002)
Sentiment	-0.004^{**}
	(0.002)
External Source	0.843^{***}
	(0.007)
Original Post	
Followers	-0.225^{***}
	(0.002)
Followees	-0.019^{***}
	(0.002)
Verified	0.154^{***}
	(0.004)
Account Age	-0.016^{***}
	(0.002)
Post Age	0.026^{***}
	(0.002)
Post Sentiment	0.000
	(0.002)
Politics	-0.026^{***}
	(0.004)
Economy	0.108^{***}
	(0.005)
Science	0.082^{***}
	(0.006)
Health	0.011^{*}
	(0.005)
Intercept	-0.153^{***}
	(0.013)
Random Effects	Included
AIC	$519{,}261$
Observations	$41{,}128$

Note: Binomial regression explains the share of helpful votes. Unit of analysis is the fact-check level ($N = 41{,}128$). Community Notes that do not link to external sources are excluded. Significance levels: $^{*}p < 0.05$, $^{**}p < 0.01$, $^{***}p < 0.001$. Standard errors are in parentheses.

Supplementary Table S9: **Regression Linking the Presence of External Sources to Helpfulness. Analysis Across Fine-Grained Topics.**

	RQ2 (with Factuality)	RQ2 (with Domain Scores)
Fact-Check		
Word Count	0.013^{***}	0.013^{***}
	(0.003)	(0.003)
Text Complexity	-0.064^{***}	-0.070^{***}
	(0.003)	(0.003)
Sentiment	0.009^{***}	0.011^{***}
	(0.002)	(0.002)
Bias in External Sources		
Bias Magnitude	-0.027^{***}	-0.030^{***}
	(0.003)	(0.003)
Factuality	0.011^{***}	
	(0.003)	
Domain Quality		-0.003
		(0.003)
Original Post		
Followers	-0.227^{***}	-0.224^{***}
	(0.003)	(0.004)
Followees	-0.025^{***}	-0.026^{***}
	(0.003)	(0.003)
Verified	0.116^{***}	0.113^{***}
	(0.007)	(0.007)
Account Age	-0.003	-0.003
	(0.003)	(0.003)
Post Age	0.005	-0.002
	(0.004)	(0.004)
Post Sentiment	-0.020^{***}	-0.019^{***}
	(0.002)	(0.002)
Political	-0.018^{***}	-0.016^{**}
	(0.005)	(0.005)
Intercept	0.777^{***}	0.777^{***}
	(0.015)	(0.015)
Random Effects	Included	Included
AIC	252,153	244,248
Observations	21,307	20,677

Note: Binomial regression explains the share of helpful votes. Unit of analysis is the fact-check level ($N = 21{,}307$). Community Notes that do not link to external sources are excluded. Significance levels: $^{*}p < 0.05$, $^{**}p < 0.01$, $^{***}p < 0.001$. Standard errors are in parentheses.

Supplementary Table S10: **Regression Linking Political Bias in External Sources to Helpfulness. RQ2 with Factuality and Domain Scores.**

	RQ3 (with Factuality)	RQ3 (with Domain Scores)
Fact-Check		
Word Count	0.013***	0.014***
	(0.003)	(0.003)
Text Complexity	−0.063***	−0.070***
	(0.003)	(0.003)
Sentiment	0.009***	0.011***
	(0.002)	(0.002)
Bias in External Sources		
Bias Magnitude	0.025**	0.016
	(0.009)	(0.008)
Bias Direction (Left)	−0.049***	−0.055***
	(0.011)	(0.011)
Bias Direction (Right)	−0.016	−0.006
	(0.015)	(0.014)
Bias Magnitude × Bias Direction (Left)	−0.082***	−0.077***
	(0.011)	(0.012)
Bias Magnitude × Bias Direction (Right)	−0.092***	−0.086***
	(0.017)	(0.016)
Factuality		
Factuality	0.041***	
	(0.010)	
Factuality × Bias Magnitude	0.010	
	(0.008)	
Factuality × Bias Direction (Left)	−0.078***	
	(0.012)	
Factuality × Bias Direction (Right)	−0.042**	
	(0.014)	
Factuality × Bias Magnitude × Bias Direction (Left)	0.075***	
	(0.011)	
Factuality × Bias Magnitude × Bias Direction (Right)	−0.028*	
	(0.011)	
Domain Quality		
Domain Quality		0.004
		(0.008)
Domain Quality × Bias Magnitude		0.005
		(0.006)
Domain Quality × Bias Direction (Left)		0.025*
		(0.011)
Domain Quality × Bias Direction (Right)		−0.024
		(0.013)
Domain Quality × Bias Magnitude × Bias Direction (Left)		−0.014
		(0.009)
Domain Quality × Bias Magnitude × Bias Direction (Right)		−0.012
		(0.008)
Intercept	0.833***	0.828***
	(0.018)	(0.018)
Original Post Controls	Included	Included
Random Effects	Included	Included
AIC	251,871	244,127
Observations	21,307	20,677

Note: Binomial regression explains the share of helpful votes. Unit of analysis is the fact-check level ($N = 21{,}307$). Community Notes that do not link to external sources are excluded. Significance levels: $^{*}p < 0.05$, $^{**}p < 0.01$, $^{***}p < 0.001$. Standard errors are in parentheses.

Supplementary Table S11: **Regression Linking Bias Direction in External Sources to Helpfulness. RQ3 with Factuality and Domain Scores.**

	RQ1
Fact-Check	
Word Count	0.048^{***}
	(0.001)
Text Complexity	-0.033^{***}
	(0.001)
Sentiment	0.018^{***}
	(0.001)
External Source	0.994^{***}
	(0.004)
Original Post	
Followers	-0.260^{***}
	(0.002)
Followees	-0.020^{***}
	(0.001)
Verified	0.094^{***}
	(0.003)
Account Age	-0.040^{***}
	(0.002)
Post Age	0.026^{***}
	(0.001)
Post Sentiment	-0.004^{***}
	(0.001)
Political	-0.050^{***}
	(0.003)
Intercept	-0.122^{***}
	(0.004)
AIC	821,890
Observations	41,128

Note: Binomial regression explains the share of helpful votes. Unit of analysis is the fact-check level ($N = 41{,}128$). Community Notes that do not link to external sources are excluded. Significance levels: $^{*}p < 0.05$, $^{**}p < 0.01$, $^{***}p < 0.001$. Standard errors are in parentheses.

Supplementary Table S12: **Regression Linking the Presence of External Sources to Helpfulness. RQ1 without Random Effects.**

	RQ2 Model (1)	RQ2 Model (2)
Fact-Check		
Word Count	0.077^{***}	0.076^{***}
	(0.002)	(0.002)
Text Complexity	-0.036^{***}	-0.036^{***}
	(0.002)	(0.002)
Sentiment	0.021^{***}	0.021^{***}
	(0.002)	(0.002)
Bias in External Sources Bias Magnitude	-0.022^{***}	-0.016^{***}
	(0.002)	(0.002)
Politics × Bias Magnitude		-0.012^{***}
		(0.004)
Original Post		
Followers	-0.248^{***}	-0.248^{***}
	(0.002)	(0.002)
Followees	-0.034^{***}	-0.034^{***}
	(0.002)	(0.002)
Verified	0.018^{***}	0.019^{***}
	(0.005)	(0.005)
Account Age	-0.021^{***}	-0.021^{***}
	(0.002)	(0.002)
Post Age	0.029^{***}	0.029^{***}
	(0.002)	(0.002)
Post Sentiment	-0.024^{***}	-0.024^{***}
	(0.002)	(0.002)
Political	-0.015^{***}	-0.014^{***}
	(0.004)	(0.004)
Intercept	0.890^{***}	0.890^{***}
	(0.004)	(0.004)
AIC	430,183	430,173
Observations	21,307	21,307

Note: Binomial regression explains the share of helpful votes. Unit of analysis is the fact-check level ($N = 21{,}307$). Community Notes that do not link to external sources are excluded. Significance levels: $^{*}p < 0.05$, $^{**}p < 0.01$, $^{***}p < 0.001$. Standard errors are in parentheses.

Supplementary Table S13: **Regression Linking Political Bias in External Sources to Helpfulness. RQ2 without Random Effects.**

	RQ3 Model (1)	RQ3 Model (2)
Fact-Check		
Word Count	0.076***	0.076***
	(0.002)	(0.002)
Text Complexity	−0.035***	−0.034***
	(0.002)	(0.002)
Sentiment	0.022***	0.022***
	(0.002)	(0.002)
Bias in External Sources		
Bias Magnitude	0.015**	0.004
	(0.005)	(0.007)
Politics × Bias Magnitude		0.017
		(0.011)
Bias Direction (Left)	0.084***	0.132***
	(0.007)	(0.010)
Bias Direction (Right)	0.109***	0.183***
	(0.009)	(0.013)
Bias Magnitude × Bias Direction (Left)	−0.202***	−0.191***
	(0.007)	(0.010)
Bias Magnitude × Bias Direction (Right)	−0.084***	−0.112***
	(0.007)	(0.010)
Politics × Bias Direction (Left)		−0.102***
		(0.014)
Politics × Bias Direction (Right)		−0.148***
		(0.018)
Politics × Bias Magnitude × Bias Direction (Left)		−0.021
		(0.015)
Politics × Bias Magnitude × Bias Direction (Right)		0.057***
		(0.014)
Original Post		
Followers	−0.248***	−0.250***
	(0.002)	(0.002)
Followees	−0.033***	−0.033***
	(0.002)	(0.002)
Verified	0.016***	0.018***
	(0.005)	(0.005)
Account Age	−0.018***	−0.018***
	(0.002)	(0.002)
Post Age	0.030***	0.030***
	(0.002)	(0.002)
Post Sentiment	−0.022***	−0.022***
	(0.002)	(0.002)
Political	−0.022***	0.049***
	(0.004)	(0.012)
Intercept	0.894***	0.860***
	(0.007)	(0.009)
AIC	428,582	428,425
Observations	21,307	21,307

Note: Binomial regression explains the share of helpful votes. Unit of analysis is the fact-check level ($N = 21{,}307$). Community Notes that do not link to external sources are excluded. Significance levels: $^{*}p < 0.05$, $^{**}p < 0.01$, $^{***}p < 0.001$. Standard errors are in parentheses.

Supplementary Table S14: **Regression Linking Bias Direction in External Sources to Helpfulness. RQ3 without Random Effects.**

	RQ1
Fact-Check	
Word Count	0.017^{**}
	(0.006)
Text Complexity	-0.029^{***}
	(0.006)
Sentiment	-0.004
	(0.006)
External Source	0.156^{***}
	(0.020)
Original Post	
Followers	0.418^{***}
	(0.007)
Followees	-0.014^{*}
	(0.006)
Verified	-0.051^{***}
	(0.014)
Account Age	-0.024^{***}
	(0.007)
Post Age	-0.065^{***}
	(0.007)
Post Sentiment	0.002
	(0.006)
Political	-0.155^{***}
	(0.012)
Intercept	3.998^{***}
	(0.022)
Random Effects	Included
AIC	416,963
Observations	41,128

Note: Binomial regression explains the share of helpful votes. Unit of analysis is the fact-check level ($N = 41{,}128$). Community Notes that do not link to external sources are excluded. Significance levels: $^{*}p < 0.05$, $^{**}p < 0.01$, $^{***}p < 0.001$. Standard errors are in parentheses.

Supplementary Table S15: **Regression Linking the Presence of External Sources to Helpfulness. RQ1 with Total Votes as the Dependent Variable.**

References

[1] Solovev, K. & Pröllochs, N. Hate Speech in the Political Discourse on Social Media: Disparities across Parties, Gender, and Ethnicity. In *Proceedings of the ACM Web Conference (WWW'22)*, 3656–3661 (2022).

[2] Yao, W., Zhang, C., Saravanan, S., Huang, R. & Mostafavi, A. Weakly-Supervised Fine-Grained Event Recognition on Social Media Texts for Disaster Management. In *Proceedings of the AAAI Conference on Artificial Intelligence (AAAI'20)*, 34, 532–539 (2020).

[3] Zhang, X. *et al.* TwHIN-Bert: A Socially-Enriched Pre-Trained Language Model for Multilingual Tweet Representations at Twitter. In *Proceedings of the ACM Conference on Knowledge Discovery and Data Mining (SIGKDD'23)*, 5597–5607 (2023).

[4] Lin, H. *et al.* High Level of Correspondence Across Different news Domain Quality Rating Sets. *PNAS Nexus* **2**, pgad286 (2023).
